# Phenomic Analysis of Chronic Granulomatous Disease Reveals More Severe Integumentary Infections in X-Linked Compared With Autosomal Recessive Chronic Granulomatous Disease

**DOI:** 10.3389/fimmu.2021.803763

**Published:** 2022-01-24

**Authors:** Timothy Lok-Hin Chiu, Daniel Leung, Koon-Wing Chan, Hok Man Yeung, Chung-Yin Wong, Huawei Mao, Jianxin He, Pandiarajan Vignesh, Weiling Liang, Woei Kang Liew, Li-Ping Jiang, Tong-Xin Chen, Xiang-Yuan Chen, Yin-Bo Tao, Yong-Bin Xu, Hsin-Hui Yu, Alta Terblanche, David Christopher Lung, Cheng-Rong Li, Jing Chen, Man Tian, Brian Eley, Xingtian Yang, Jing Yang, Wen Chin Chiang, Bee Wah Lee, Deepti Suri, Amit Rawat, Anju Gupta, Surjit Singh, Wilfred Hing Sang Wong, Gilbert T. Chua, Jaime Sou Da Rosa Duque, Kai-Ning Cheong, Patrick Chun-Yin Chong, Marco Hok-Kung Ho, Tsz-Leung Lee, Wanling Yang, Pamela P. Lee, Yu Lung Lau

**Affiliations:** ^1^ Department of Paediatrics and Adolescent Medicine, The University of Hong Kong, Queen Mary Hospital, Hong Kong, Hong Kong SAR, China; ^2^ Department of Immunology, Beijing Children’s Hospital, Capital Medical University, National Center for Children’s Health, Beijing, China; ^3^ Department of Respiratory Medicine, Beijing Children’s Hospital, Capital Medical University, National Center for Children’s Health, Beijing, China; ^4^ Allergy & Immunology Unit, Department of Paediatrics, Advanced Pediatrics Centre, Postgraduate Institute of Medical Education and Research, Chandigarh, India; ^5^ Department of Paediatrics and Adolescent Medicine, The University of Hong Kong-Shenzhen Hospital, Shenzhen, China; ^6^ Paediatric Immunology Service, KK Hospital, Singapore, Singapore; ^7^ Children’s Hospital of Chongqing Medical University, Chongqing, China; ^8^ Shanghai Children’s Medical Center, School of Medicine, Shanghai Jiao Tong University, Shanghai, China; ^9^ Department of Allergy, Immunology and Rheumatology, Guangzhou Children’s Hospital, Guangdong, China; ^10^ Guangzhou Women and Children’s Medical Center, Guangzhou, China; ^11^ Department of Paediatrics, National Taiwan University Children’s Hospital, Taipei, Taiwan; ^12^ Paediatric Gastroenterology and Hepatology Unit, University of Pretoria, Pretoria, South Africa; ^13^ Department of Pathology, Queen Elizabeth Hospital/Hong Kong Children’s Hospital, Hong Kong, Hong Kong SAR, China; ^14^ Department of Nephrology, Shenzhen Children’s Hospital, Shenzhen, China; ^15^ Department of Hematology/Oncology, Key Laboratory of Pediatric Hematology & Oncology Ministry of Health, Shanghai Children’s Medical Center, Shanghai Jiao Tong University School of Medicine, Shanghai, China; ^16^ Department of Tuberculosis, Nanjing Chest Hospital, Nanjing, China; ^17^ Department of Paediatrics and Child Health, University of Cape Town and Red Cross War Memorial Children’s Hospital, Cape Town, South Africa; ^18^ Department of Paediatrics, Yong Loo Lin School of Medicine, National University of Singapore, Singapore, Singapore; ^19^ Khoo Teck Puat-National University Children’s Medical Institute, National University Health System, Singapore, Singapore; ^20^ Hong Kong Children’s Hospital, Hong Kong, Hong Kong SAR, China; ^21^ Virtus Medical, Hong Kong, Hong Kong SAR, China

**Keywords:** chronic granulomatous disease (CGD), inborn error of immunity (IEI), human phenotype ontology (HPO), phenome, genetics

## Abstract

**Background:**

Chronic granulomatous disease (CGD) is an inborn error of immunity (IEI), characterised by recurrent bacterial and fungal infections. It is inherited either in an X-linked (XL) or autosomal recessive (AR) mode. Phenome refers to the entire set of phenotypes expressed, and its study allows us to generate new knowledge of the disease. The objective of the study is to reveal the phenomic differences between XL and AR-CGD by using Human Phenotype Ontology (HPO) terms.

**Methods:**

We collected data on 117 patients with genetically diagnosed CGD from Asia and Africa referred to the Asian Primary Immunodeficiency Network (APID network). Only 90 patients with sufficient clinical information were included for phenomic analysis. We used HPO terms to describe all phenotypes manifested in the patients.

**Results:**

XL-CGD patients had a lower age of onset, referral, clinical diagnosis, and genetic diagnosis compared with AR-CGD patients. The integument and central nervous system were more frequently affected in XL-CGD patients. Regarding HPO terms, perianal abscess, cutaneous abscess, and elevated hepatic transaminase were correlated with XL-CGD. A higher percentage of XL-CGD patients presented with BCGitis/BCGosis as their first manifestation. Among our CGD patients, lung was the most frequently infected organ, with gastrointestinal system and skin ranking second and third, respectively. *Aspergillus* species, *Mycobacterium bovis*, and *Mycobacteirum tuberculosis* were the most frequent pathogens to be found.

**Conclusion:**

Phenomic analysis confirmed that XL-CGD patients have more recurrent and aggressive infections compared with AR-CGD patients. Various phenotypic differences listed out can be used as clinical handles to distinguish XL or AR-CGD based on clinical features.

## Introduction

Chronic granulomatous disease (CGD) is an inborn error of immunity (IEI) that is characterised by recurrent infections caused by catalase-positive bacteria and fungi, such as *Staphylococcus aureus* and *Aspergillus* species (1). It is estimated that the prevalence of CGD is 1 in 250,000 live births among Europeans and Americans (2, 3). CGD arises from the loss of function of one of the proteins forming the NADPH oxidase complex, which generates reactive oxygen species, i.e., superoxide radicals and hydrogen peroxide for intracellular bacteria and fungi killing in phagocytes (4). Currently, there is one X-linked (XL) and five autosomal recessive (AR) forms of CGD. The gene responsible for XL-CGD is *CYBB*, and the other five genes responsible for AR-CGD are *CYBA*, *NCF1*, *NCF2*, *NCF4*, and *CYBC1*. Frequently affected organs and systems include the lung, skin, lymph node, and liver (3). Patients may suffer from pneumonia and deep and superficial abscesses. CGD patients usually present with lymphadenopathy and hepatosplenomegaly on physical examination (5).

In our study, we focus on the phenomic analysis of CGD. Phenomics stands for the acquisition of high-dimensional phenotypic data on an organism scale. Study of phenomics is usually incorporated with the study of genomics and environment so that we can know various factors which might possibly influence the complex traits displayed. Compared with genomics, phenomics is much more sophisticated and is much more difficult to be characterised (6). For this study, we use Human Phenotype Ontology (HPO) terms to describe the phenomic abnormalities. The HPO project was publicised in 2008 and provides an ontology of annotations (7), i.e., HPO terms, to describe phenotypic abnormalities encountered by clinicians. The HPO currently contains over 13,000 terms arranged in a simple class-subclass relationship such that various specific terms belong to a subclass of a parent term. The aim of the HPO system is to offer a computational bridge between genome biology and clinical medicine, as well as enabling the integration of phenotypic information across various scientific tools for clinical diagnosis and research purposes (7). Due to advancement in technology, there are more external tools available for genomic discovery project and diagnostic research. For genomic projects, HPO terms are used to filter out the list of candidate genes to be tested from whole genome sequencing using Exomiser, Phevor, or PhenIX (8, 9). External algorithms such as Phenomizer and Phenolyzer can compute clinical phenotype data written in HPO terms to give out possible differential diagnoses. Phenomizer is an external tool which utilises HPO terms to report phenotypic abnormalities. It yields a list of differential diagnoses of the patient based on the HPO data inputted by a clinician (10). However, for most IEI which are included in the genotypic classification of the International Union of the Immunological Societies, a full HPO phenomic data is still lacking currently which requires contributions from different clinical immunologists (11, 12). Therefore, it is paramount to generate a phenome of IEI for a reference for the differential diagnosis tool. This can provide sufficient information for them to diagnose future patients by importing HPO terms identified by medical practitioners.

Currently, numerous case series about CGD patients have already been published but none of them has used HPO terms to represent their phenome. The phenomic data on CGD patients stored in Phenomizer database may not be accurate as well due to insufficient phenomic analysis. As a result, the main aim of this project is to observe and create a phenome for CGD patients in our case series. We also attempted to identify the main differences of the phenotypic data between XL-CGD and AR-CGD patients. The phenome of the respective XL or AR-CGD patients may also be sent to various external tools which use HPO terms for analysis such as Phenomizer and Exomiser so as to provide a reference for diagnosis and genetic analysis of suspected CGD patients. Significant differences are listed out to help clinicians to differentiate between them clinically.

## Materials and Methods

### Patient Source and Selection

The APID network is an IEI referral network established in 2009 by The University of Hong Kong, which acts as a platform to offer e-consultation and free genetic testing for IEI. There are over 100 member centres across Asia and Africa. From 2003 to 2017, 117 CGD patients referred from 18 centres were successfully genetically diagnosed and were included in this case series.

### Data Collection

Clinical records and laboratory results of CGD patients, provided by their referring doctors at the time of request for genetic testing, are archived in the APID network. Patient data, including demographics, family history, age of clinical milestones, infection, and genetic results were recorded.

### Phenomic Data

HPO (October 2020 version) terms, which describe individual phenotypic abnormalities in a hierarchical framework of organs, were applied for performing the phenomic analysis. Two researchers first reviewed the laboratory reports and clinical notes and then suitable HPO terms were selected from the HPO browser http://www.human-phenotype-ontology.org to describe the phenotypic abnormalities displayed. In the end, a HPO phenotypic profile for each CGD patient was generated. The HPO phenotypic profile consisting of all HPO terms which can be manifested from the clinical records was selected. No negated HPO terms, i.e., no specific phenotype was manifested in the clinical record, were used in our study. Discrepancies for the final HPO phenotypic profile were discussed and modified. Only the highest class of HPO terms in the hierarchial framework, i.e., systems affected and the most specific HPO terms were computed for detection of any significant correlation between individual phenotypic abnormality and mode of inheritance.

### Genetics Data

Genetic analysis was performed using genomic DNA extracted from peripheral blood. Genomic DNA was sent to our research laboratory and the candidate genes, i.e., *CYBB*, *CYBA*, *NCF1*, *NCF2*, and *NCF4*, were tested by using Sanger sequencing on the basis of clinical likelihood in the research laboratory of the Department of Paediatrics and Adolescent Medicine, The University of Hong Kong. Pathogenicity of the targeted gene mutation is re-evaluated in accordance with the diagnostic interpretation guidelines published by the American Academy of Allergy, Asthma & Immunology (AAAAI) PID working group in 2020 (13).

### APID Network Questionnaire

A questionnaire was also distributed online to APID network member centres across Asia and Africa. Questions regarding the availability of care for CGD patients in APID network member centres, i.e., diagnosis, laboratory tests, and treatment of CGD patients were asked. A total of 20 responses were recorded.

### Ethics Approval

This research project is approved by The University of Hong Kong/Hospital Authority Hong Kong West Cluster Institutional Review Board.

### Statistical Analysis

For descriptive statistics, all ages of clinical milestones were expressed in median and range (year). Univariate analysis was performed by independent-samples Mann-Whitney *U* test to evaluate the difference between XL-CGD and AR-CGD. First manifestation, HPO terms, and system affected are presented in the form of heat map and expressed in percentages. Fisher’s exact test was used in analysis to determine the correlation between categorical phenotypes with the genotype.

## Results

### Demographics Data

**Figure 1:**
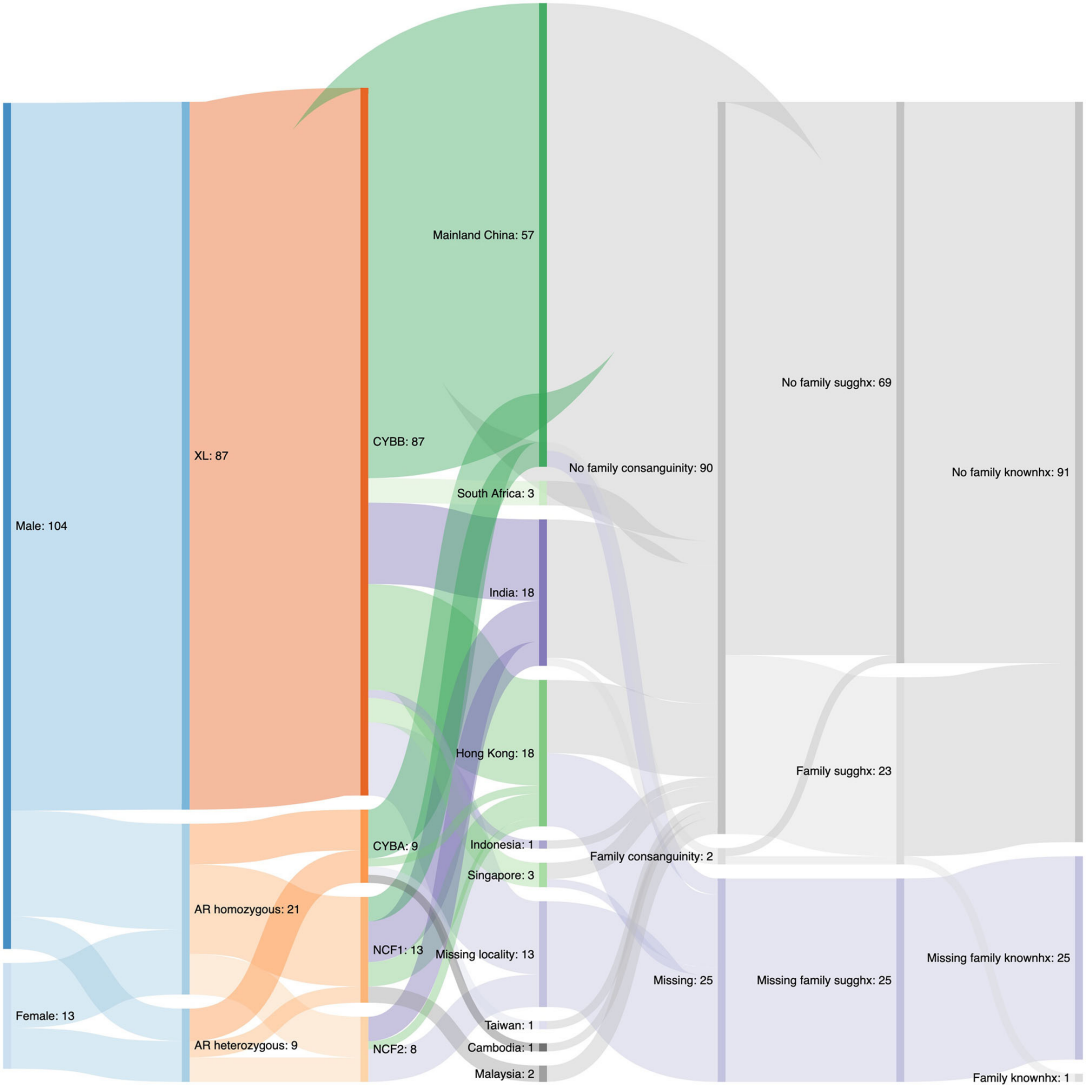
A Sankey diagram describing demographics data of the CGD case series. XL, X-linked; AR, autosomal recessive; sugghx, suggested history; knownhx, known history.

The demographics data of our CGD case series was displayed in the Sankey diagram in **Figure 1**. Of the 117 patients, 104 (88.9%) were male and 13 (11.1%) were female. XL-CGD was seen in 87 (74.4%) patients, while the remaining belonged to AR-CGD group. Out of the 30 AR CGD patients, 9 (30.0%) of them were found to have mutations in *CYBA* by Sanger Sequencing, 13 (43.3%) of them were found to have GT deletion in *NCF1* by GeneScan^®^ and 9 (30.0%) of them were found to have mutations in *NCF2* by Sanger Sequencing, with one of them have concurrent *CYBA* and *NCF2* mutations diagnosed. In our case series, more than half of the patients (56.7%) came from mainland China, with India (17.3%) and Hong Kong (17.3%) ranking second. Other patients either came from South-east Asia or South Africa. Details about consanguinity and family history were only available for 92 out of 117 patients, with 90 (97.8%) having no family consanguinity and 2 (2.2%) with family consanguinity. Moreover, out of the 92 patients, 69 (75.0%) have no suggestive family history and 23 (25.0%) have suggestive family history. Only 1 of them has an elder brother with known CGD.

### Genetics Data

The genetic mutations of 117 CGD patients, with 3 patients reporting 2 unphased variants, are shown in **Table 1**. The commonest gene implicated was *CYBB*, with no diagnosed *NCF4* CGD patients. In total, 87 of them had mutations identified in *CYBB* gene. For the remaining 30 AR-CGD patients, 20 of them had homozygous mutations while only 1 of them was confirmed with compound heterozygous. Four of them had 2 unphased heterozygous mutations found in a recessive gene and five of them had 1 heterozygous mutation found in a recessive gene.

**Table 1:** Genetics data of CGD patients.

Patient no	Sex	Gene	Mode of inheritance	cDNA position	Codon change	Mutation type	Pathogenicity#	References
1	M	CYBB	X-linked hemizygous	LRG_53t1:c.252G>A	p.Ala84=, predicted aberrant splicing	Splice site	**Pathogenic (PVS1, PS3)**	(14), ClinVar: VCV000010933.6
2	M	CYBB	X-linked hemizygous	LRG_53t1:c.252G>A	p.Ala84=, predicted aberrant splicing	Splice site	**Pathogenic (PVS1, PS3)**	(14), ClinVar: VCV000010933.6
3	M	CYBB	X-linked hemizygous	LRG_53t1:c.742dup	p.Ile248Asnfs*36	Frameshift with premature stop codon	**Pathogenic (PVS1, PM2, PP4') **	(14)
4	M	CYBB	X-linked hemizygous	LRG_53t1:c.613T>A	p.Phe205Ile	Missense	**Likely pathogenic (PM2, PM1, PP4')**	(15, 16),
5	M	CYBB	X-linked hemizygous	LRG_53t1:c.1498G>C	p.Asp500His	Missense	**Pathogenic (PS3, PM1, PM2, PP4')**	(14)
6	M	CYBB	X-linked hemizygous	LRG_53t1:c.1555G>T	p.Glu519*	Nonsense	**Pathogenic (PVS1, PS2, PM2, PP4')**	(15, 16),
7	M	CYBB	X-linked hemizygous	LRG_53t1:c.646_648del	p.Phe216del	Deletion	**Pathogenic (PS3, PM2, PP4', PP5, PP2) **	(17)
8	M	CYBB	X-linked hemizygous	LRG_53t1:c.1025T>A	p.Leu342Gln	Missense	**Likely pathogenic (PM2, PP4', PP5, PP2)**	(16)
9	M	CYBB	X-linked hemizygous	LRG_53t1:c.713del	p.Val238Glyfs*4	Frameshift with premature stop codon	**Pathogenic (PVS1, PM2, PP4')**	(16, 18),
10	M	CYBB	X-linked hemizygous	LRG_53t1:c.1327del	p.Trp443Glyfs*59	Frameshift with premature stop codon	**Pathogenic (PVS1, PM2, PP4')**	(16, 18),
11	M	CYBB	X-linked hemizygous	LRG_53t1:c.935T>A	p.Met312Lys	Missense	**Likely pathogenic (PM2, PM1, PP4',)**	(16, 18),
12	M	CYBB	X-linked hemizygous	LRG_53t1:c.1437C>A	p.Tyr479*	Nonsense	**Pathogenic (PVS1, PM2, PP4')**	(16, 18),
13	M	CYBB	X-linked hemizygous	LRG_53t1:c.253-1G>A (RT-PCR: LRG_53t1:c.253_266del)	RT-PCR show aberrant splicing, Cys85Serfs*13	Splice site	**Pathogenic (PVS1, PM2, PP4')**	(11)
14	M	CYBB	X-linked hemizygous	LRG_53t1:c.46-1G>C	Predicted aberrant splicing	Splice site	**Pathogenic (PVS1, PM2, PP4')**	Not reported
15	M	CYBB	X-linked hemizygous	LRG_53t1:c.577T>C	p.Ser193Pro	Missense	**Pathogenic (PS3, PM2, PM1, PP4') **	(14, 18),
16	M	CYBB	X-linked hemizygous	LRG_53t1:c.868C>T	p.Arg290*	Nonsense	**Pathogenic (PVS1, PM2, PP4')**	(14, 18),
17	M	CYBB	X-linked hemizygous	LRG_53t1:c.1315-2A>C	/	Splice site	**Pathogenic (PVS1, PM2, PP4')**	(16, 19),
18	M	CYBB	X-linked hemizygous	LRG_53t1:c.1713A>T	p.*571Tyrext*8	Elongation	**Likely pathogenic (PM2, PP4', PM1)**	Not reported
19	M	CYBB	X-linked hemizygous	LRG_53t1:c.77_78del	p.Phe26Cysfs*8	Frameshift with premature stop codon	**Pathogenic (PVS1, PM2, PP4')**	(7)
20	M	CYBB	X-linked hemizygous	LRG_53t1:c.469C>T	p.Arg157*	Nonsense	**Pathogenic (PVS1, PM2, PP4')**	(20, 21),
21	M	CYBB	X-linked hemizygous	LRG_53t1:c.857_867del	p.Val286Alafs*58	Frameshift with premature stop codon	**Pathogenic (PVS1, PM2, PP4')**	Not reported
22	M	CYBB	X-linked hemizygous	LRG_53t1:c.676C>T	p.Arg226*	Nonsense	**Pathogenic (PVS1, PM2, PP4')**	(19), ClinVar: VCV000010929.5
23	M	CYBB	X-linked hemizygous	LRG_53t1:c.742del	p.Ile248Serfs*7	Frameshift with premature stop codon	**Pathogenic (PVS1, PM2, PP4')**	(22)
24	M	CYBB	X-linked hemizygous	LRG_53t1:c.1234G>A	p.Gly412Arg	Missense	**Likely pathogenic (PM2, PP4', PM1)**	Not reported
25	M	CYBB	X-linked hemizygous	LRG_53t1:c.674+608_1587-1407del (EX7-EX11del)	p.Arg226Profs*14	Large deletion	**Pathogenic (PVS1, PM2, PP4')**	Not reported
26	M	CYBB	X-linked hemizygous	LRG_53t1:c.804+2T>C	Predicted aberrant splicing	Splicing	**Pathogenic (PVS1, PM2, PP4')**	(16, 21),
27	M	CYBB	X-linked hemizygous	LRG_53t1:c.1583C>G	p.Pro528Arg	Missense	**Likely pathogenic (PM2, PP4', PM1)**	Not reported
28	M	CYBB	X-linked hemizygous	LRG_53t1:c.626A>G	p.His209Arg	Missense	**Pathogenic (PS3, PM2, PP4', PM1)**	(23)
29	M	CYBB	X-linked hemizygous	LRG_53t1:c.45+1G>A	Predicted aberrant splicing	Splice site	**Pathogenic (PVS1, PM2, PP4')**	(24)
30	M	CYBB	X-linked hemizygous	LRG_53t1:c.1164_1166delinsATC	p.Asp388_Gly389delinsGluSer	In-frame deletion/insertion	**Likely pathogenic (PM2, PP4', PM5)**	Not reported
31	M	CYBB	X-linked hemizygous	LRG_53t1:c.1014C>A	p.His338Gln	Missense	**Likely pathogenic (PM2, PP4', PM1) **	(16, 25),
32	M	CYBB	X-linked hemizygous	LRG_53t1:c.1399G>T	p.Glu467*	Nonsense	**Pathogenic (PVS1, PM2, PP4')**	(16, 22),
33	M	CYBB	X-linked hemizygous	LRG_53t1:c.1016C>A	p.Pro339His	Missense	**Pathogenic (PS3, PM2, PP4', PM1)**	(17)
34	M	CYBB	X-linked hemizygous	EX11-EX13del	Predicted no protein expression	Large deletion	**Pathogenic (PVS1, PM2, PP4')**	Not reported
35	M	CYBB	X-linked hemizygous	LRG_53t1:c.911C>G; EX11-EX13del	p.Pro304Arg and predicted protein truncation	Missense and large deletion	**Pathogenic (PVS1, PM2, PP4')**	(25, 26),
36	M	CYBB	X-linked hemizygous	LRG_53t1:c.925G>A	p.Glu309Lys	Missense	**Pathogenic (PS3, PM2, PP4', PM1)**	(17)
37	M	CYBB	X-linked hemizygous	LRG_53t1:c.1150_1151+2delAAGT	Predicted aberrant splicing	Splice site	**Pathogenic (PVS1, PM2, PP4')**	(27)
38	M	CYBB	X-linked hemizygous	LRG_53t1:c.376T>C	p.Cys126Arg	Missense	**Pathogenic (PS2, PS3, PM2, PP4', PM1)**	(25)
39	M	CYBB	X-linked hemizygous	LRG_53t1:c.1332del	p.Cys445Alafs*57	Frameshift with premature stop codon	**Pathogenic (PVS1, PM2, PP4')**	Not reported
40	M	CYBB	X-linked hemizygous	LRG_53t1:c.469C>T	p.Arg157*	Nonsense	**Pathogenic (PVS1, PM2, PP4')**	(20, 21),
41	M	CYBB	X-linked hemizygous	LRG_53t1:c.70_72del	p.Phe24del	Deletion	**Likely pathogenic (PM2, PP4', PM1)**	(22)
42	M	CYBB	X-linked hemizygous	LRG_53t1:c.676C>T	p.Arg226*	Nonsense	**Pathogenic (PVS1, PM2, PP4')**	(19), ClinVar: VCV000010929.5
43	M	CYBB	X-linked hemizygous	LRG_53t1:c.665A>G	p.His222Arg	Missense	**Pathogenic (PS3, PM2, PP4', PM1)**	(19)
44	M	CYBB	X-linked hemizygous	LRG_53t1:c.1244C>T	p.Pro415Leu	Missense	**Likely pathogenic (PM2, PP4', PM1)**	(19),
45	M	CYBB	X-linked hemizygous	LRG_53t1:c.1313del	p.Lys438Argfs*64	Frameshift with premature stop codon	**Pathogenic (PVS1, PM2, PP4')**	(16, 21),
46	M	CYBB	X-linked hemizygous	LRG_53t1:c.1328G>A	p.Trp443*	Nonsense	**Pathogenic (PVS1, PM2, PP4')**	(16, 19),
47	M	CYBB	X-linked hemizygous	LRG_53t1:c.126_130delinsTTTC	p.Arg43Phefs*18	Frameshift with premature stop codon	**Pathogenic (PVS1, PM2, PP4')**	(16)
48	M	CYBB	X-linked hemizygous	LRG_53t1:c.868C>T	p.Arg290*	Nonsense	**Pathogenic (PVS1, PM2, PP4')**	(18, 28),
49	M	CYBB	X-linked hemizygous	LRG_53t1:c.674+6T>C	Predicted aberrant splicing	Splicing	**Pathogenic (PVS1, PM2, PP4')**	Not reported
50	M	CYBB	X-linked hemizygous	LRG_53t1:c.1619_1626dup	p.Ala543Lysfs*7	Frameshift with premature stop codon	**Pathogenic (PVS1, PM2, PP4')**	Not reported
51	M	CYBB	X-linked hemizygous	LRG_53t1:c.1038del	p.Glu347Argfs*39	Frameshift with premature stop codon	**Pathogenic (PVS1, PM2, PP4')**	Not reported
52	M	CYBB	X-linked hemizygous	LRG_53t1:c.141+3A>T	Predicted aberrant splicing	Splicing	**Pathogenic (PVS1, PM2, PP4')**	Not reported
53	M	CYBB	X-linked hemizygous	LRG_53t1:c.271C>T	p.Arg91*	Nonsense	**Pathogenic (PVS1, PM2, PP4')**	(21, 29),
54	M	CYBB	X-linked hemizygous	LRG_53t1:c.1151+1G>A	Predicted aberrant splicing	Splice site	**Pathogenic (PVS1, PM2, PP4')**	Not reported
55	M	CYBB	X-linked hemizygous	LRG_53t1:c.1314+2T>G	Predicted aberrant splicing	Splice site	**Pathogenic (PVS1, PM2, PP4')**	Not reported
56	M	CYBB	X-linked hemizygous	LRG_53t1:c.45+1G>A	Predicted aberrant splicing	Splice site	**Pathogenic (PVS1, PM2, PP4')**	Not reported
57	M	CYBB	X-linked hemizygous	LRG_53t1:c.469C>T	p.Arg157*	Nonsense	**Pathogenic (PVS1, PM2, PP4')**	(21)
58	M	CYBB	X-linked hemizygous	LRG_53t1:c.742dup	p.Ile248Asnfs*36	Frameshift with premature stop codon	**Pathogenic (PVS1, PM2, PP4')**	(21)
59	M	CYBB	X-linked hemizygous	LRG_53t1:c.1548G>C	p.Trp516Cys	Missense	**Likely pathogenic (PM2, PP4', PP5, PP2)**	(16)
60	M	CYBB	X-linked hemizygous	LRG_53t1:c.252G>A	p.A84=	Splice site	**Pathogenic (PVS1, PS3)**	(30), ClinVar: VCV000010933.6
61	M	CYBB	X-linked hemizygous	LRG_53t1:c.123C>G	p.Tyr41*	Nonsense	**Pathogenic (PVS1, PM2, PP4')**	Not reported
62	M	CYBB	X-linked hemizygous	LRG_53t1:c.676C>T	p.Arg226*	Nonsense	**Pathogenic (PVS1, PM2, PP4')**	(19), ClinVar: VCV000010929.5
63	M	CYBB	X-linked hemizygous	LRG_53t1:c.868C>T	p.Arg290*	Nonsense	**Pathogenic (PVS1, PM2, PP4')**	(18, 29),
64	M	CYBB	X-linked hemizygous	LRG_53t1:c.675-1G>T	Predicted aberrant splicing	Splice site	**Pathogenic (PVS1, PM2, PP4')**	Not reported
65	M	CYBB	X-linked hemizygous	LRG_53t1:c.-65C>T	/	Promoter	**Likely pathogenic (PS3, PM2, PP4')**	(31)
66	M	CYBB	X-linked hemizygous	LRG_53t1:c.1022C>T	p.Thr341Ile	Missense	**Pathogenic (PS3, PM2, PP4', PP5, PP2)**	(16, 32),
67	M	CYBB	X-linked hemizygous	LRG_53t1:c.742dup	p.Ile248Asnfs*36	Frameshift with premature stop codon	**Pathogenic (PVS1, PM2, PP4')**	(14, 21),
68	M	CYBB	X-linked hemizygous	LRG_53t1:c.252G>A	p.A84=	Splice site	**Pathogenic (PVS1, PS3)**	(30), ClinVar: VCV000010933.6
69	M	CYBB	X-linked hemizygous	LRG_53t1:c.724_725del	p.Thr242Serfs*3	Frameshift with premature stop codon	**Pathogenic (PVS1, PM2, PP4')**	(33)
70	M	CYBB	X-linked hemizygous	Exon 8-13 deletion	/	Large deletion	**Pathogenic (PVS1, PM2, PP4')**	Not reported
71	M	CYBB	X-linked hemizygous	LRG_53t1:c.714_715insTA	p.His239Tyrfs*4	Frameshift with premature stop codon	**Pathogenic (PVS1, PM2, PP4')**	(34)
72	M	CYBB	X-linked hemizygous	LRG_53t1:c.45+1G>C	Predicted aberrant splicing	Splice site	**Pathogenic (PVS1, PM2, PP4')**	Not reported
73	M	CYBB	X-linked hemizygous	LRG_53t1:c.84G>A	p.Trp28*	Nonsense	**Pathogenic (PVS1, PM2, PP4')**	(35)
74	M	CYBB	X-linked hemizygous	LRG_53t1:c.1154T>G	p.Ile385Arg	missense	**Pathogenic (PS3, PM2, PP4', PP5, PP2)**	(16, 32),
75	M	CYBB	X-linked hemizygous	LRG_53t1:c.1075G>A	p.Gly359Arg	missense	**Pathogenic (PS3, PM2, PP4', PP5, PP2)**	(14, 32),
76	M	CYBB	X-linked hemizygous	LRG_53t1:c.217C>T	p.Arg73*	Nonsense	**Pathogenic (PVS1, PM2, PP4')**	(35)
77	M	CYBB	X-linked hemizygous	LRG_53t1:c.1322_1324del	p.Phe441del	In-frame deletion/insertion	**Uncertain significance (PM2, PP4')**	Not reported
78	M	CYBB	X-linked hemizygous	LRG_53t1:c.141+1_141+2del	Predicted aberrant splicing	Splice site	**Pathogenic (PVS1, PM2, PP4')**	Not reported
79	M	CYBB	X-linked hemizygous	LRG_53t1:c.1546T>C	p.Trp516Arg	Missense	**Pathogenic (PS3, PM2, PP4', PP5, PP2)**	(36)
80	M	CYBB	X-linked hemizygous	LRG_53t1:c.676C>T	p.Arg226*	Nonsense	**Pathogenic (PVS1, PM2, PP4')**	(19), ClinVar: VCV000010929.5
81	M	CYBB	X-linked hemizygous	LRG_53t1:c.676C>T	p.Arg226*	Nonsense	**Pathogenic (PVS1, PM2, PP4')**	(19), ClinVar: VCV000010929.5
82	M	CYBB	X-linked hemizygous	LRG_53t1:c.722_726delTAACA	p.Ile241Serfs*3	Frameshift with premature stop codon	**Pathogenic (PVS1, PM2, PP4')**	(19)
83	M	CYBB	X-linked hemizygous	LRG_53t1:c.388C>T	p.Arg130*	Nonsense	**Pathogenic (PVS1, PM2, PP4')**	(21)
84	M	CYBB	X-linked hemizygous	LRG_53t1:c.45+2delT	Predicted aberrant splicing	Splice site	**Pathogenic (PVS1, PM2, PP4')**	Not reported
85	M	CYBB	X-linked hemizygous	LRG_53t1:c.1414G>A	p.Gly472Ser	Missense	**Likely pathogenic (PM2, PP4', PP5, PP2)**	(25)
86	M	CYBB	X-linked hemizygous	LRG_53t1:c.985T>C	p.Cys329Arg	Missense	**Pathogenic (PM2, PP4', PP5, PP2)**	(37)
87	M	CYBB	X-linked hemizygous	LRG_53t1:c.868C>T	p.Arg290*	Nonsense	**Pathogenic (PVS1, PM2, PP4')**	(18)
88	F	CYBA	Compound heterozygous	LRG_52t1:c.70G>A	p.Gly24Arg	Missense	**Pathogenic (PS3, PM2, PP4', PP5, PP2)**	(38–40)
				LRG_52t1:c.204-2A>G	predicted aberrant splicing	splice site	**Pathogenic (PVS1, PM2, PP4')**	
89	M	CYBA	Heterozygous	LRG_52t1:c.418G>A	p.Glu140Lys	Missense	**Uncertain significance (PM2, PP4')**	ClinVar: VCV000966844.1
90	F	CYBA	2 heterozygous (not known if compound heterozygous)	LRG_52t1:c.7C>T	p.Gln3*	Nonsense	**Pathogenic (PVS1, PM2, PP4')**	(40, 41)
				LRG_52t1:c.59-2A>G	Predicted aberrant splicing	Splice site	**Pathogenic (PVS1, PM2, PP4')**	
91	M	CYBA	Homozygous	LRG_52t1:c.7C>T	p.Gln3*	nonsense	**Pathogenic (PVS1, PM2, PP4')**	(40, 41)
92	F	CYBA	2 heterozygous (not known if compound heterozygous)	LRG_52t1:c.7C>T	p.Gln3*	Nonsense	**Pathogenic (PVS1, PM2, PP4')**	(40, 41)
				LRG_52t1:c.129-23_129-5del	Predicted aberrant splicing	Splice site	**Pathogenic (PVS1, PM2, PP4')**	
93	F	CYBA	Homozygous	LRG_52t1:c.7C>T	p.Gln3*	Nonsense	**Pathogenic (PVS1, PM2, PP4')**	(40, 41)
94	F	CYBA	Homozygous	LRG_52t1:c.482_498delinsC	p.Glu162Leufs*3	Frameshift with premature stop codon	**Pathogenic (PVS1, PM2, PP4')**	(41)
95	F	CYBA	Homozygous	LRG_52t1:c.371C>T	p.Ala124Val	Missense	**Pathogenic (PS3, PM2, PP4', PP5, PP2)**	(40)
96	M	CYBA	Homozygous	LRG_52t1:c.205G>T	p.Gly69*	Nonsense	**Pathogenic (PVS1, PM2, PP4')**	(42–45), ClinVar: VCV000002248.10
		NCF2	Heterozygous	LRG_88t1:c.1183C>T	p.Arg395Trp	Missense	**Uncertain significance due to conflicting interpretations@**	
97	M	NCF1	Homozygous	LRG_87t1:c.75_76del	p.Tyr26Hisfs*26	Frameshift with premature stop codon	**Pathogenic (PVS1, PM2, PP4')**	(46)
98	F	NCF1	Homozygous	LRG_87t1:c.75_76del	p.Tyr26Hisfs*26	Frameshift with premature stop codon	**Pathogenic (PVS1, PM2, PP4')**	(46)
99	M	NCF1	Homozygous	LRG_87t1:c.75_76del	p.Tyr26Hisfs*26	Frameshift with premature stop codon	**Pathogenic (PVS1, PM2, PP4')**	(46)
100	F	NCF1	Homozygous	LRG_87t1:c.75_76del	p.Tyr26Hisfs*26	Frameshift with premature stop codon	**Pathogenic (PVS1, PM2, PP4')**	(46)
101	F	NCF1	Heterozygous	LRG_87t1:c.75_76del	p.Tyr26Hisfs*26	Frameshift with premature stop codon	**Pathogenic (PVS1, PM2, PP4')**	(46)
102	M	NCF1	Homozygous	LRG_87t1:c.75_76del	p.Tyr26Hisfs*26	Frameshift with premature stop codon	**Pathogenic (PVS1, PM2, PP4')**	(46)
103	M	NCF1	Homozygous	LRG_87t1:c.75_76del	p.Tyr26Hisfs*26	Frameshift with premature stop codon	**Pathogenic (PVS1, PM2, PP4')**	(46)
104	F	NCF1	Homozygous	LRG_87t1:c.75_76del	p.Tyr26Hisfs*26	Frameshift with premature stop codon	**Pathogenic (PVS1, PM2, PP4')**	(46)
105	M	NCF1	Homozygous	LRG_87t1:c.75_76del	p.Tyr26Hisfs*26	Frameshift with premature stop codon	**Pathogenic (PVS1, PM2, PP4')**	(46)
106	M	NCF1	Homozygous	LRG_87t1:c.75_76del	p.Tyr26Hisfs*26	Frameshift with premature stop codon	**Pathogenic (PVS1, PM2, PP4')**	(46)
107	M	NCF1	Homozygous	LRG_87t1:c.75_76del	p.Tyr26Hisfs*26	Frameshift with premature stop codon	**Pathogenic (PVS1, PM2, PP4')**	(46)
108	F	NCF1	Homozygous	LRG_87t1:c.75_76del	p.Tyr26Hisfs*26	Frameshift with premature stop codon	**Pathogenic (PVS1, PM2, PP4')**	(46)
109	F	NCF1	Heterozygous	LRG_87t1:c.75_76del	p.Tyr26Hisfs*26	Frameshift with premature stop codon	**Pathogenic (PVS1, PM2, PP4')**	(46)
110	M	NCF2	Homozygous	LRG_88t1:c.835_836del	p.Thr279Glyfs*16	Frameshift with premature stop codon	**Pathogenic (PVS1, PM2, PP4')**	(30, 47–49)
111	M	NCF2	2 heterozygous (not known if compound heterozygous)	LRG_88t1:c.1099C>T	p.Gln367*	Frameshift with premature stop codon	**Pathogenic (PVS1, PM2, PP4')**	(16, 48)
				LRG_88t1:c.1179-2A>T	Predicted aberrant splicing	Splice site	**Pathogenic (PVS1, PM2, PP4')**	
112	M	NCF2	Homozygous	LRG_88t1:c.835_836del	p.Thr279Glyfs*16	Frameshift with premature stop codon	**Pathogenic (PVS1, PM2, PP4')**	(30, 47–49)
113	M	NCF2	Heterozygous	LRG_88t1:c.1183C>T	p.Arg395Trp	Missense	**Uncertain significance due to conflicting interpretations@**	(43–45), ClinVar: VCV000002248.10
114	M	NCF2	Heterozygous	LRG_88t1:c.1183C>T	p.Arg395Trp	Missense	**Uncertain significance due to conflicting interpretations@**	(43–45), ClinVar: VCV000002248.10
115	F	NCF2	Homozygous	LRG_88t1:c.835_836del	p.Thr279Glyfs*16	Frameshift with premature stop codon	Pathogenic (PVS1, PM2, PP4')	(49)
116	M	NCF2	Homozygous	LRG_88t1:c.501+1_501+8del	Predicted aberrant splicing	Splice site	**Pathogenic (PVS1, PM2, PP4')**	Not reported
117	M	NCF2	Homozygous	LRG_88t1:c.835_836del	p.Thr279Glyfs*16	Frameshift with premature stop codon	Pathogenic (PVS1, PM2, PP4')	(49)

Mutation nomenclature is made according to Locus Reference Genome (LRG). Mutation pathogenicity curation is made according to the AAAAI guideline 2020.

^*^PP4’ denotes an increase of pathogenicity to “moderate” level, as suggested by the AAAAI PID Genetics guidance.

Criteria for classifying pathogenic variants as abbreviated by the ACMG Standards and Guidelines for the Interpretation of Sequence Variants: https://www.ncbi.nlm.nih.gov/pmc/articles/PMC4544753/.

It is considered pathogenic allele in NCBI SNP database with conflicting interpretation in pathogenicity (ClinVar) in 2017.

All AR-CGD patients with *NCF1* mutations have documented GT deletion. Four patients among our CGD case series had a large deletion mutation in *CYBB*. There were 24 novel mutations identified in our patients, including 23 mutations in *CYBB* and 1 mutation in *NCF2*. Pathogenicity of these unreported mutations was determined by using the AAAAI guidelines in 2020 (13). Among 117 CGD patients, 99 of the CGD patients have pathogenic variants, 13 of them have likely pathogenic variants, and 5 of them have variants with uncertain significance.

### Clinical Characteristics of XL and AR-CGD

The ages of clinical milestones of our CGD patients are displayed in **Table 2**. Ages of onset, referral, clinical diagnosis, and genetic diagnosis correlated with the mode of inheritance. Median age of onset correlated with the mode of inheritance (*p* = 0.01), with XL-CGD (0.2 years) lower than that of AR-CGD (0.4 years). Median age of referral to an immunology unit of XL-CGD (0.8 years) is also significantly lower than that of AR-CGD (3.5 years) and was shown to be significantly related with the mode of inheritance (*p* = 0.009). Median age at clinical diagnosis of XL-CGD (1.4 years) is younger than that of AR-CGD (4.8 years) with a strong correlation between XL or AR (*p* = 0.017). The same result was also demonstrated for the age of genetic diagnosis (*p* = 0.004) with XL-CGD patients showing a lower median age (2.2 years) compared with AR-CGD patients (4.8 years).

**Table 2:** Comparison between clinical characteristics between XL-CGD and AR-CGD using Fisher’s exact test.

Parameter	XL (range) (*n* = 67)	AR (range) (*n* = 23)	*p*-value
Median age of onset	0.2 (0–5) (*n* = 66)	0.4 (0–13.3) (*n* = 19)	0.01^**^
Median age of referral to immunology centre	0.75 (0–14) (*n* = 64)	3.5 (0.1–26.6) (*n* = 18)	0.009^**^
Median age at clinical diagnosis	1.4 (0–14) (*n* = 58)	4.8 (0.2–26.7) (*n* = 13)	0.017
Median age at genetic diagnosis	2.2 (0.1–14.8) (*n* = 70)	4.7 (0.9–26.7) (*n* = 19)	0.004
Median delay in referral to immunology centre	0.3 (0–12) (*n* = 59)	0.25 (0–12) (*n* = 14)	0.794
Median delay in diagnosis of CGD	0 (−0.3–6.2) (*n* = 53)	0.3 (0–1) (*n* = 8)	0.375

XL, X-linked; AR, autosomal recessive; CGD, chronic granulomatous disease. ^**^p < 0.01.

**Figure 2:**
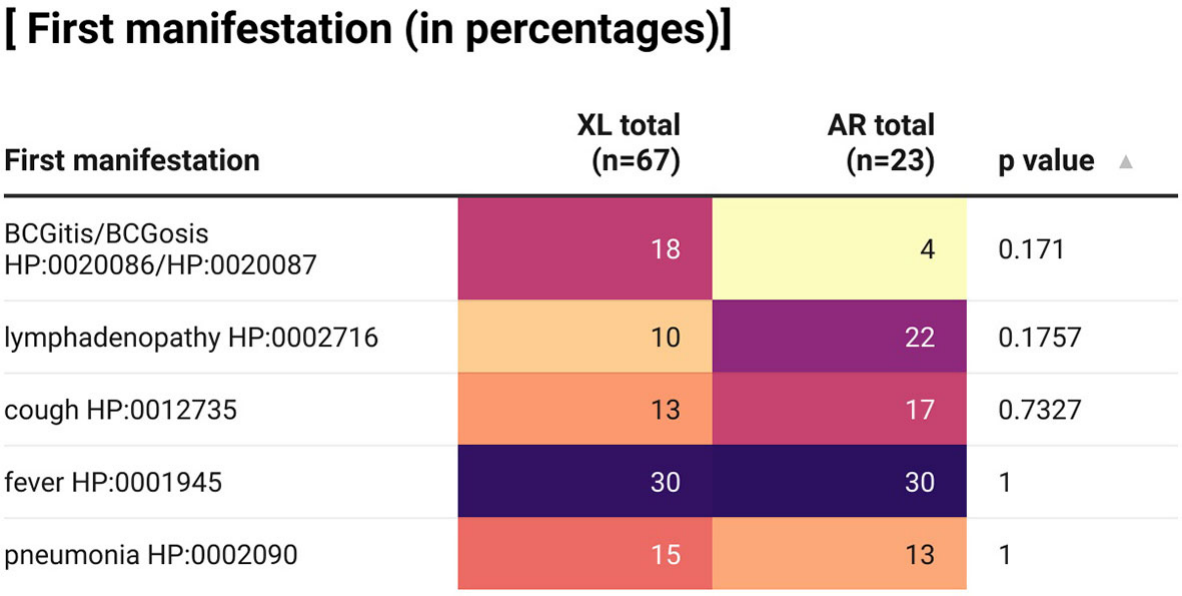
A heat map describing percentages of CGD patients in our case series with certain first manifestations according to their clinical records. XL, X-linked; AR, autosomal recessive; BCG, Bacillus Calmette–Guérin. Fisher’s exact test is used, *p*-value <0.05 is significant. This graph is created by using the app Datawrapper.

First manifestations of XL and AR-CGD patients in our case series were displayed in **Figure 2**. Only the 5 most common first manifestations were included in the heat map, namely fever, BCGitis/BCGosis, pneumonia, cough, and lymphadenopathy, where generalized lymphadenopathy and cervical lymphadenopathy or no specific location regarding the lymphadenopathy were all categorized here. As shown in the figure, there is no significant association between the mode of inheritance and the respective first manifestation. However, it could be seen that a higher percentage of XL-CGD (18%) has BCGitis/BCGosis as their first manifestation compared with AR-CGD (4%) while a higher proportion of AR CGD (22%) has lymphadenopathy as their first manifestation compared with XL CGD (10%).

### Infection Profile

**Figure 3:**
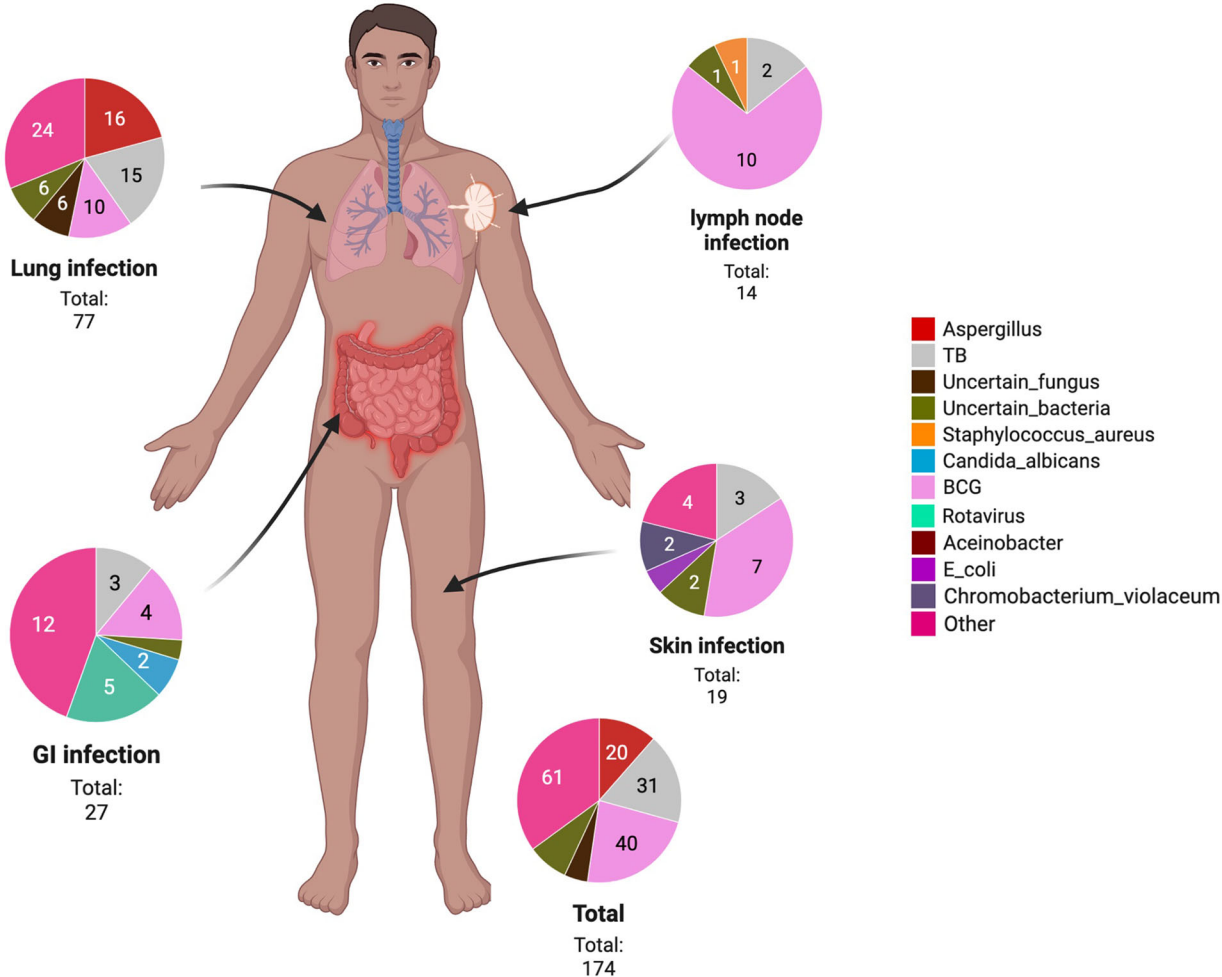
A body map showing the infection location of pathogens in our CGD case series. GI, gastrointestinal; TB, *Mycobacterium tuberculosis*; *E. coli*, *Escherichia coli*; BCG, Bacillus Calmette–Guérin.

Results for microbiological testing ordered based on clinical suspicion of infections were tallied, and cultured microorganisms from various infections and the 3 top locations where they were cultured are reported in **Figure 3**. However, the infectious etiology may not be established in every CGD case as the culture of the pathogen may not be performed or the culture reports were negative due to the use of antibiotics or antifungals prior to the culture. As shown above, a significant portion of infection was caused by uncertain bacteria or fungi. In general, the most common pathogens isolated are the *Mycobacteirum bovis* (*n* = 40), *Mycobacteirum tuberculosis* (*n* = 31), and *Aspergillus* species (*n* = 23). *M. bovis* was mostly isolated in the lungs, lymph nodes and the skin. *M. tuberculosis* was isolated in multiple systems or organs including the lungs, gastrointestinal system, skin, or disseminated infection while *Aspergillus* species were cultured mostly in the lungs. The most frequent locations of infections are the lungs (*n* = 77), gastrointestinal system (*n* = 27), and the skin (*n* = 19). Other locations of infection include the heart (*n* = 1), upper respiratory tract (*n* = 1), central nervous system (*n* = 1), ear (*n* = 2), neck (*n* = 2), spleen (*n* = 6), liver (*n* = 3), lymph node (*n* = 14), bone (*n* = 7), and disseminated infection (*n* = 3). Among the 77 episodes of infection affecting the lungs, *Aspergillus* species (*n* = 18) and *M. tuberculosis* (*n* = 15) were most often isolated, causing mostly pneumonia and bronchitis. Among the 23 episodes of infection affecting the gastrointestinal system, *Rotavirus* (*n* = 5) was most often isolated from the alimentary canal, causing mostly diarrhoea, vomiting, and enterocolitis. Other infectious etiologies causing hepato-splenomegaly and hepatic and splenic abscesses were not included here. Among the 19 episodes of infections affecting the skin, *M. bovis* (*n* = 7) is the most commonly isolated, causing cutaneous abscesses most of the time.

### Phenomic Analysis Between XL and AR-CGD

**Figure 4:**
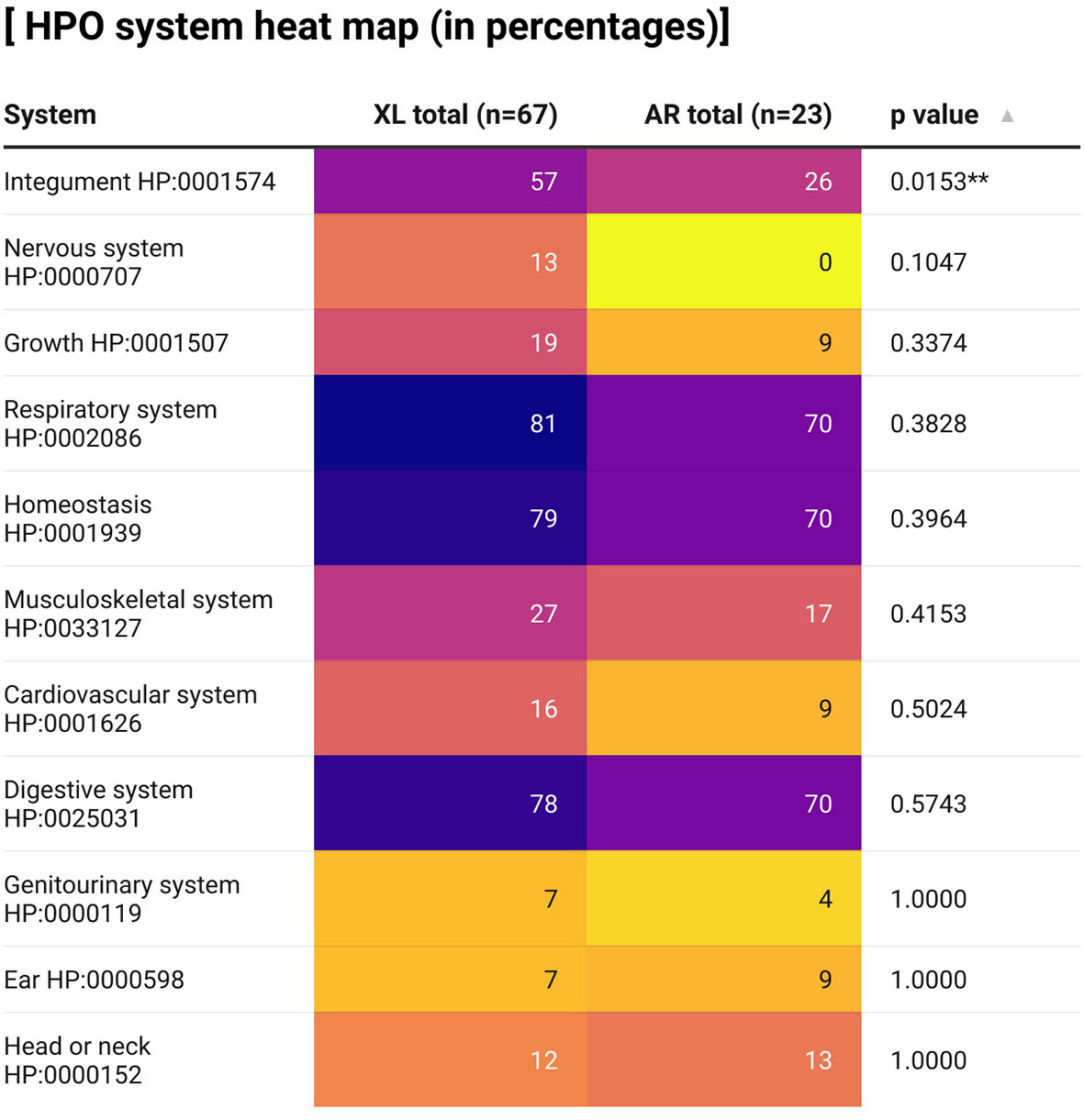
A heat map describing percentages of CGD patients in our case series in which their organ systems are affected. HPO, Human Phenotype Ontology. ^**^ *p* < 0.01. This graph is created by using the app Datawrapper.

Regarding the phenomic analysis, we only included 90 out of 117 patients as only these 90 patients had sufficient clinical information provided. The affected systems of XL and AR-CGD patients are displayed in **Figure 4** in the form of a heat map. In general, more XL-CGD patients are affected compared with AR-CGD patients in terms of the systems. Immune system was not shown in the heat map because all CGD patients had their immune system affected. As shown above, the most frequently affected systems of both XL and AR-CGD patients were the respiratory system, homeostasis system, and digestive system, respectively. A univariate analysis was performed to determine the correlation between various systems and their respective genotypes by using Fisher’s exact test. The integumentary system is significantly associated between the mode of inheritance (*p* = 0.0153), with more XL-CGD patients (57%) affected compared with AR-CGD patients (26%). In addition, more XL-CGD patients (13%) have their nervous system affected compared with no AR-CGD patients showing such manifestation. Although it is not statistically significant, it is still an interesting phenomenon to be reported.

**Figure 5:**
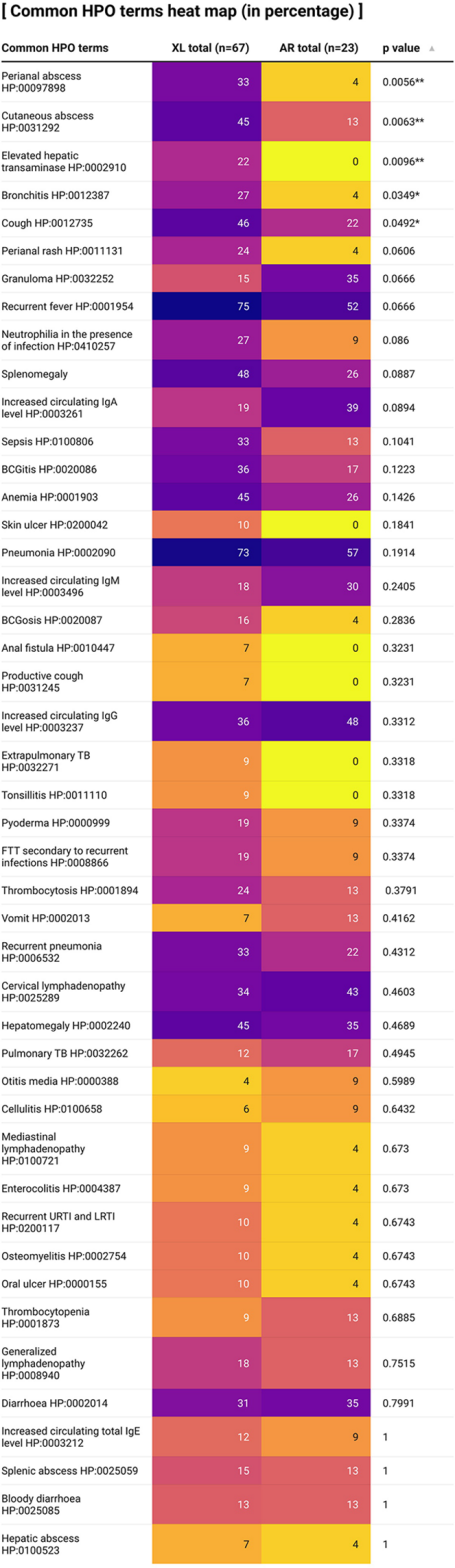
A heat map describing percentages of CGD patients in our case series where their clinical records displayed certain phenotypic abnormalities. Fisher’s exact test is used for statistical analysis (Only more than 5% of patients describing certain HPO term is recorded). HPO, Human Phenotype Ontology. Note: ^**^ *p* < 0.01; ^*^ *p* < 0.05. This graph is created by using the app Datawrapper.

Phenotypic abnormalities of CGD patients are displayed in **Figure 5** in the form of a heat map as shown above. More than 200 HPO terms describing phenotypic abnormalities were recorded in our CGD case series but only HPO terms which were manifested by more than 10% of patients would be included in the heat map. In general, more HPO terms were displayed in XL-CGD patients compared with AR-CGD patients. Among all the HPO terms recorded, both recurrent fever and pneumonia are the most frequent HPO terms identified with more than 70% of XL-CGD patients and 50% of AR-CGD patients showing this phenotypic abnormality. Other more common phenotypic abnormalities include hepatosplenomegaly, cutaneous abscess, and anaemia.

A total of 5 HPO terms were shown to be correlated with XL or AR inheritance by Fisher’s exact test with more XL-CGD patients showing that specific phenotypic abnormality. Perianal abscess, cutaneous abscess and elevated hepatic transaminase are strongly correlated with the mode of inheritance (*p* < 0.001). Bronchitis and cough are correlated with the mode of inheritance as well (*p* < 0.05).

### APID Network Questionnaire Regarding the Care of CGD Patients

**Figure 6:**
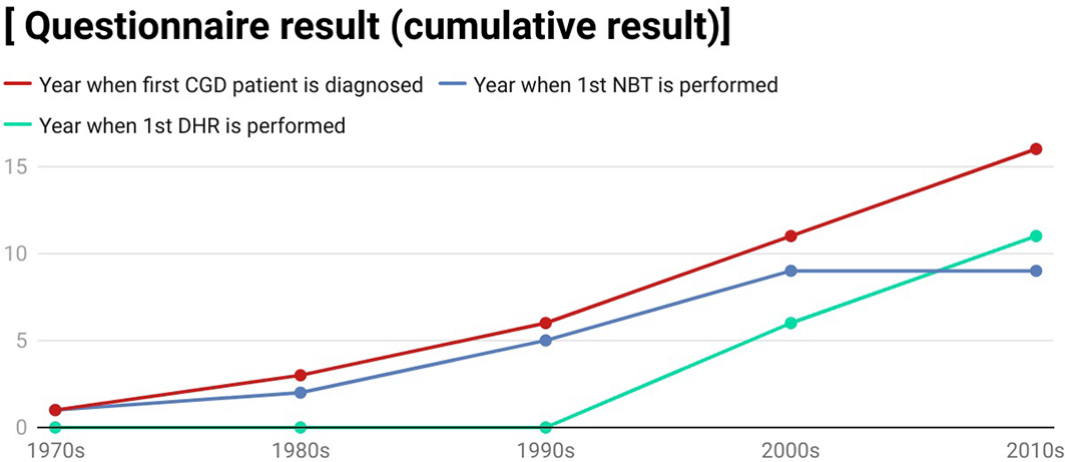
A line graph showing the questionnaire result which we deliver to various immunology centres. This graph is created by using the app Datawrapper.

The results of the questionnaire which was delivered to 20 APID network members are shown in **Figure 6**. As displayed, there were 16 centres who had diagnosed CGD in their clinics and most of them diagnosed their first CGD patients in the 1990s to 2010s. Only 9 APID network members have performed nitroblue tetrazolium test (NBT) after their establishment. APID network members diagnosed CGD by using NBT test initially with all of the 9 clinics performing the first NBT test before 2010s. However, starting from 1990s, APID network members started to use dihydrorhodamine (DHR) cytometry assay as well. In total, 11 APID network members have performed DHR cytometry assay in their clinics during the 1990s to 2010s.

## Discussion

Our study revealed that XL-CGD and AR-CGD patients had some phenotypic differences through phenomic analysis. XL-CGD patients had their integument and the central nervous system more frequently affected. XL-CGD patients were shown to have perianal abscess, cutaneous abscess, and elevated hepatic transaminase more often as well. More XL-CGD patients presented with BCGitis/BCGosis as their first manifestation.

In our study, the most significant finding is that the integument system is more frequently affected among XL-CGD patients than AR-CGD patients. The reason behind such finding is due to more frequent perianal abscess, perianal rash, and cutaneous abscess reported among XL-CGD patients in the phenomic analysis, as it was observed in previous publications (3, 50–52). However, from reports in India and China, there is no statistical difference between XL and AR-CGD patients with episodes of superficial abscess (30, 41). Another interesting observation reported in our case series is that 13% of XL-CGD patients, but none of the AR-CGD patients had a central nervous system (CNS) abnormality. Common abnormalities under CNS include upper motor neuron dysfunction, headache, spinal cord compression, choroid plexus cyst, and unusual CNS infection, including CNS aspergillosis. The frequency of CNS aspergillosis only accounts for less than 5% in overall infections, and it has been shown that there is no significant association between the genotype and CNS aspergillosis in previous literature (53, 54). Further investigations need to be done to see whether these CNS and integumentary abnormalities are primary defects or complications of CGD or unrelated with CGD. Nevertheless, these new findings might be useful clinical handles for clinical immunologist to distinguish between XL and AR-CGD. Whenever clinicians observe some redflags, i.e., more frequent cutaneous or perianal abscesses or CNS abnormalities among CGD patients, they should suspect XL-CGD and perform targeted gene Sanger sequencing and DHR as soon as possible to confirm the diagnosis.

In addition to more frequent perianal and cutaneous abscesses seen in XL-CGD patients, a higher frequency of elevated hepatic transaminase was noted among XL-CGD patients in phenomic analysis. Previous literature has shown that abnormal liver enzymes level is common among CGD patients, occurring with at least one episode among 73% of the patients (55). However, no study has been done to correlate with the mode of inheritance and elevated hepatic transaminase. It has been hypothesised that XL-CGD patients had hepatosplenomegaly, liver abscesses, and BCGosis more often, which is a common cause of abnormal liver enzymes shown in previous literature (55). Therefore, clinicians can use the frequency of elevated hepatic transaminase to help differentiate between XL-CGD and AR-CGD.

Another notable finding in this case series is that more XL-CGD patients presented with BCGitis/BCGosis as first manifestation compared with AR-CGD patients. It has been documented that CGD patients are more prone to disseminated or local BCG infection due to defective intracellular mycobacterial killing mechanism (18, 56). BCG-related disease has been documented as a common first sign of CGD but in-depth study between genotypes of CGD has not been done. BCGitis is seen more commonly in countries where BCG vaccination is included in universal vaccination programme like mainland China, Iran, and Latin America as shown in **Table 3** (67). It has been hypothesised that patients with XL-CGD had poorer control of BCG as compared with AR-CGD and hence physicians can recognise the BCG-related disease more often as their first manifestation.

**Table 3:** A summary of findings regarding the differences between previous case series and this case series.

CGD case series (year published)	Region	Total no of patients (XL/AR)	Percentage of XL patients	Percentage of male patients	Median or mean age of onset in years (XL/AR)	Median or mean age of diagnosis in years (XL/AR)	Most common infectious etiology	Most common infection location	Frequency of BCGitis (XL/AR)	Mortality rate	First manifestation
This case series	Asia and Africa	118	75%	89%	(0.2/0.4)	(1.4/4.8)	*M. bovis*	Lungs	(36%/17%)	/	fever
Rawat et al. (30)	India	236	44%	73%	0.7 (0.5/1.0)	2.0 (1.0/2.5)	*Aspergillus*	Lungs	/	60%	/
Blancas-Galicia et al. (57)	Mexico	93	77%	88%	0.3	2.5	*S. aureus*	Lungs	58%	40%	/
Gao et al. (41)	China	159	89%	89%	/	1.3	*M. tuberculosis* and *Aspergillus*	Lungs	/	43%	/
Zhou et al. (22)	China	169	89%	89%	0.1 (0.1/0.1)	0.7 (0.7/0.8)	*S. aureus*	Lungs	59%	37%	recurrent fever
Oliveira-Junior et al. (58)	Latin America	71	75%	82%	2.0 (1.8/2.8)	4.4 (3.6/8.2)	*S. aureus*	Lungs	30%	0%	/
Bortoletto et al. (52)	USA	27	70%	85%	/	3 (2.1/5.3)	*S. aureus*	Lungs	/	15%	/
Rawat et al. (29)	India	17	41%	88%	0.8 (0.3/1.3)	3 (1/3.5)	*Aspergillus*	Lungs	/	35%	/
Marciano et al. (59)	USA	268	69%	/	/	5.4 (3.2/11)	*Aspergillus fumigatus*	Lungs	/	17%	/
Koker et al. (60)	Turkey	89	41%	72%	/	4.2 (2.7/5.2)	*Aspergillus*	Lungs	23%	10%	/
Fattahi et al. (61)	Iran	93	13%	62%	(0.5/1.7)	(0.9/5.8)	*Aspergillus fumigatus*	Lungs	56%	10%	severe lymphadenopathy
van den Berg et al. (2)	Europe	429	67%	82%	/	(4.9/8.8)	*S. aureus*	Lungs	8%	20%	/
Jones et al. (50)	UK	94	81%	93%	/	2.7	*Aspergillus*	Lungs	/	12%	/
Wolach et al. (51)	Israel	38	29%	68%	/	/	*S. aureus*	Lungs	/	26%	recurrent pneumonia
Martire et al. (62)	Italy	60	65%	97%	0.6	2.5 (2/5.5)	*Aspergillus*	Lungs	/	13%	pneumonia
Agudelo-Florez et al. (63)	Latin America	14	/	64%	/	/	*/*	Lungs	0%	/	/
Carnide et al. (64)	Brazil	18	70%	89%	/	1.1	/	Lungs	/	33.00%	pneumonia
Liese et al. (65)	Germany	39	82%	95%	0.7 (0.3/1.1)	5.4 (3.8/13.6)	*S. aureus*	Lungs	/	20%	lymphadenitis
Winkelstein et al. (3)	USA	368	76%	86%	/	(3.0/7.8)	*Aspergillus*	Lungs	/	18%	/
Hasui et al. (66)	Japan	221	/	88%	/	/	*/*	Lungs	/	23%	/

XL, X-linked; AR, autosomal recessive; CGD, chronic granulomatous disease.

The main limitation of our study is that the clinical data provided to the APID network might be insufficient. Some of the CGD patients from our study were genetically diagnosed 20 years ago, during which the awareness and understanding of CGD was still inadequate in many countries, leading to an underreporting of CGD patients with atypical features. The authors do not have full access to the complete medical records of the CGD patients and hence some major phenotypic data of the CGD patients in our case series may be missed. Microbiological culture tests were not performed in some cases, leading to omissions in our infection profile. *Staphylococcus aureus*, for example, has been reported to be the most common pathogen causing skin abscesses in previous CGD case series but was not shown in our study. In addition, the clinical data provided to us were only up to the time when the patient was clinically diagnosed with CGD, and hence no follow-up clinical data could be computed and analysed. As a result, survival and death analysis cannot be done. As NBT or DHR assays were not always available and our cases came from many centres with different testing methodologies (68), therefore the functional phenotype of residual reactive oxygen species production could not be analysed in our case series. Since there were only 23 AR-CGD cases with sufficient clinical information, there might not be enough power resulting in false-negative results in our phenomic analysis comparing between AR and XL-CGD.

In conclusion, more severe integument infections, CNS, and hepatic enzyme abnormalities were observed in XL-CGD patients compared with AR-CGD patients. A summary of key findings regarding the differences between previous case series and this case series is presented in **Table 3**. Whenever clinicians identify such phenomic features among our children suspected to have IEI, they should suspect a diagnosis of XL-CGD and perform DHR as soon as possible. This can help speed up the diagnostic process and hence start prophylactic treatment as well as offering targeted genetic testing.

## Data Availability Statement

The datasets presented in this article are not readily available because of ethical restrictions. Requests to access the datasets should be directed to lauylung@hku.hk.

## Ethics Statement

The studies involving human participants were reviewed and approved by Hospital Authority Hong Kong West Cluster-University of Hong Kong Institutional Review Board. Written informed consent to participate in this study was provided by the participants’ legal guardian/next of kin.

## Author Contributions

YL conceptualised the study. YL and DL designed the study. K-WC and C-YW performed genetic study. TC, HY, K-WC, and DL curated mutations. TC and DL phenotyped the patients, analysed data, and penned the manuscript. Other authors referred patients and provided clinical care and clinical data. All authors critically reviewed the manuscript. All authors contributed to the article and approved the submitted version.

## Funding

The work is funded by the Society for Relief of Disabled Children and Jeffrey Modell Foundation.

## Conflict of Interest

The authors declare that the research was conducted in the absence of any commercial or financial relationships that could be construed as a potential conflict of interest.

The handling editor declared a past co-authorship with the authors DL, HM, SS, PPL and YLL.

## Publisher’s Note

All claims expressed in this article are solely those of the authors and do not necessarily represent those of their affiliated organizations, or those of the publisher, the editors and the reviewers. Any product that may be evaluated in this article, or claim that may be made by its manufacturer, is not guaranteed or endorsed by the publisher.
